# Supplementary material for: Infants’ First Solid Foods: Impact on Gut Microbiota Development in Two Intercontinental Cohorts
Source: Nutrients. 2021 Jul 30;13(8):2639. doi: 10.3390/nu13082639 (PMC8400337; doi:10.3390/nu13082639)
Supplement: Supplementary file 1 [file nutrients-13-02639-s001.zip › nutrients-1317413-supplementary.pdf]

Infants' First Solid Foods: Impact on Gut Microbiota Development in Two Intercontinental Cohorts.

Supplementary Materials

Table S1: Results of the linear mixed effects models for observed richness and the demographic/nutritional variables of interest for the two cohorts. Multivariable models are adjusted for estimated total energy intake (kcal/d). Diversity scores are further adjusted for age (d), age at introduction and GBS prophylaxis.

	Baby, Food & Mi				LucKi-Gut			
	Univariable		Multivariable		Univariable		Multivariable	
	Estimate	Sig.	Estimate	Sig.	Estimate	Sig.	Estimate	Sig.
Age (d)	0.239	**0.0233**	-	-	0.496	p < 0.01	-	-
Sex	5.34	n.s.	-	-	22.279	p < 0.05	-	-
Total energy (kcal/d)	0.042	**0.0159**	-	-	0.125	p < 0.01	-	-
Carbohydrates (kcal/d)	0.052	n.s.	0.091	n.s.	0.110	p < 0.1	-0.182	n.s.
Fiber (g/d)	0.38	n.s.	-0.00039	n.s.	3.306	p < 0.05	-0.032	n.s.
Protein (kcal/d)	0.059	n.s.	0.052	n.s.	1.603	p < 0.05	0.882	n.s.
Fat (kcal/d)	-0.0010	n.s.	-0.086	n.s.	0.274	p < 0.05	0.166	n.s.
Food Diversity	1.11	n.s.	1.43	p < 0.1	1.049	n.s.	0.082	n.s.
Pre-/Probiotic Diversity	1.19	n.s.	1.52	**p < 0.05**	2.638	n.s.	2.559	n.s.
Food Diversity (/d)	0.057	n.s.	0.083	n.s.	0.641	p < 0.05	0.260	n.s.
Pre-/Probiotic Diversity (/d)	0.66	n.s.	0.87	n.s.	0.140	p < 0.1	0.036	n.s.

Table S2: Results of the linear mixed effects models for Shannon alpha diversity and the demographic/nutritional variables of interest for the two cohorts. Multivariable models are adjusted for total energy intake (kcal/d), age (d), age introduction and GBS prophylaxis.

	Baby, Food & Mi				LucKi-Gut			
	Univariable		Multivariable		Univariable		Multivariable	
	Estimate	Sig.	Estimate	Sig.	Estimate	Sig.	Estimate	Sig.
Age (d)	0.0086	**0.0269**	-	-	0.001	n.s.	-	-
Sex	0.0018	n.s.	-	-	0.443	n.s.	-	-
Total energy (kcal/d)	0.0024	**0.00095**	-	-	0.001	n.s.	-	-
Carbohydrates (kcal/d)	0.0057	**0.0121**	0.003	n.s.	-0.001	n.s.	-0.001	n.s.
Fiber (g/d)	0.082	**0.0193**	0.113	**p < 0.05**	0.026	n.s.	-0.009	n.s.

Protein (kcal/d)	0.011	$p < 0.1$	-0.012	n.s.	0.015	n.s.	0.011	n.s.
Fat (kcal/d)	0.0026	$p < 0.1$	-0.001	n.s.	0.001	n.s.	0.001	n.s.
Food Diversity	0.014	n.s.	0.025	n.s.	-0.006	n.s.	0.006	n.s.
Pre-/Probiotic Diversity	0.017	n.s.	0.028	$p < 0.1$	0.031	n.s.	0.056	n.s.
Food Diversity (/d)	-0.0031	n.s.	-0.0085	n.s.	0.002	n.s.	-0.001	n.s.
Pre-/Probiotic Diversity (/d)	-0.018	n.s.	0.00066	n.s.	0.002	n.s.	0.002	n.s.

Table S3: Results of DESeq analysis for all bacterial ASVs significantly associated with fiber intake (g/d), daily food diversity score and daily pre-/probiotic diversity score in the Baby, Food & Mi study.

**Fiber intake (g/d)**			
ASV	Base Mean	log_2folddifference	adj. p-value
Clostridium_sensu_stricto_1 ASV 23	643.5	-15.2	< 0.00001
Bacteroides ASV 71	188.0	-12.1	< 0.00001
Enterobacteriaceae ASV 119	68.4	-11.7	< 0.00001
Bacteroides ASV 16	114.0	-11.3	< 0.00001
Bacteroides ASV 87	43.7	-9.8	< 0.00001
Shimwellia ASV 98	23.5	-9.6	< 0.00001
Bacteroides ASV 14	14.4	-8.2	< 0.00001
Atopobiaceae ASV 121	76.8	-6.5	< 0.00001
Bacteroides ASV 37	2025.4	5.5	< 0.00001
Escherichia/Shigella ASV 4	4666.1	-4.5	< 0.00001
Bifidobacterium ASV 15	483.4	-3.2	< 0.00001
**Food Diversity Score (/d)**			
ASV	Base Mean	log_2folddifference	adj. p-value
Veillonella ASV 99	273.0	-23.6	< 0.00001
Veillonella ASV 26	151.7	-22.8	< 0.00001
Citrobacter ASV 92	92.1	-21.7	< 0.00001
Bacteroides ASV 107	45.9	-21.2	< 0.00001
Bacteroides ASV 48	33.9	-20.7	< 0.00001
Bacteroides ASV 102	24.5	-20.3	< 0.00001
Citrobacter ASV 72	20.9	-20.1	< 0.00001
Veillonella ASV 61	10.1	-19.0	< 0.00001
Parabacteroides ASV 20	81.5	-0.8	< 0.00001
Bacteroides ASV 103	947.8	-0.6	0.000302
Bacteroides ASV 163	291.3	-0.5	0.00180
Bacteroides ASV 54	262.5	-0.5	0.00193
Parabacteroides ASV 151	105.7	-0.5	0.00726
Phascolarctobacterium ASV 79	68.7	-0.5	0.00862
Parabacteroides ASV 12	24.2	-0.4	0.0178
Escherichia/Shigella ASV 4	585.5	-0.4	0.0262
Lactobacillus ASV 6	3494.7	-0.4	0.000204
Klebsiella ASV 32	223.4	-0.4	0.0178
Bifidobacterium ASV 10	4438.7	-0.4	0.0435

Bifidobacterium ASV 3	17836.2	0.3	0.0435
Clostridioides ASV 45	58.6	0.3	0.0465
Veillonella ASV 147	60.3	0.3	0.0465
Shimwellia ASV 98	5.9	0.4	0.0435
Enterococcus ASV 64	69.0	0.4	0.0435
Enterobacteriaceae ASV 119	6.3	0.4	0.0435
Parabacteroides ASV 133	84.2	0.4	0.0112
Atopbociaceae ASV 121	70.5	0.6	< 0.00001
Bacteroides ASV 87	40.4	0.9	< 0.00001
Bacteroides ASV 128	53.8	1.1	< 0.00001
Bacteroides ASV 37	1176.8	1.3	< 0.00001
**Pre-/Probiotic Diversity Score (/d)**			
ASV	Base Mean	log_2folddifference	adj. p-value
Veillonella ASV 26	44.6	-18.8	< 0.00001
Bacteroides ASV 48	33.9	-15.2	< 0.00001
Parabacteroides ASV 12	24.2	-11.7	< 0.00001
Citrobacter ASV 92	18.3	-11.6	< 0.00001
Phascolarctobacterium ASV 79	15.8	-11.4	< 0.00001
Veillonella ASV 61	10.1	-11.1	< 0.00001
Bacteroides ASV 54	9.3	-11.1	< 0.00001
Bacteroides ASV 107	7.2	-10.9	< 0.00001
Parabacteroides ASV 20	81.5	-2.0	0.0273
Klebsiella ASV 32	223.4	-1.7	0.0484
Lactobacillus ASV 6	305.8	-1.4	0.00734
Bifidobacterium ASV 8	6225.4	1.3	0.0319
Bacteroides ASV 40	336.1	1.8	0.0487
Enterococcus ASV 64	69.0	1.9	0.0487
Veillonella ASV 96	41.0	2.0	0.0299
Escherichia/Shigella ASV 4	585.5	2.1	0.0213
Atopobiaceae ASV 121	70.5	2.2	0.0213
Bifidobacterium ASV 10	4409.6	5.1	< 0.00001
Bacteroides ASV 186	39.7	5.4	< 0.00001
Bacteroides ASV 37	1176.8	5.5	< 0.00001
Bacteroides ASV 128	53.8	5.6	< 0.00001
Parabacteroides ASV 133	68.9	5.8	< 0.00001
Bacteroides ASV 71	122.2	6.0	< 0.00001
Bacteroides ASV 87	40.4	6.2	< 0.00001

Table S4: Results of DESeq analysis for all bacterial ASVs that were significantly associated with fiber intake (g/d) and daily food diversity score in the LucKi-Gut study. No bacterial ASVs were associated with the daily pre-/probiotic diversity score. *: these ASVs are also among the 10 most abundant ASVs in the LucKi-Gut study.

**Fiber intake (g/d)**			
ASV	Base Mean	log$_2$folddifference	adj. p-value
Proteus ASV 7	66.0	12.9	< 0.00001
Clostridium neonatale ASV 9*	532.9	-2.2	0.00353
Clostridium difficile ASV 4	61.4	-3.6	0.00004
Bifidobacterium ASV 33*	730.4	-3.4	0.0163
Clostridium neonatale ASV 22	47.7	-24.1	< 0.00001
**Food Diversity Score (/d)**			
ASV	Base Mean	log$_2$folddifference	adj. p-value
Bacteroides ASV 170	36.2	-3.1	< 0.00001
Bacteroides ASV 183	191.1	-1.2	< 0.00001
Sutterella ASV 22	28.6	-0.8	0.00038
Bacteroides ASV 155	54.4	-1.0	0.00090
Escherichia coli ASV 20	35.9	-0.9	0.00364

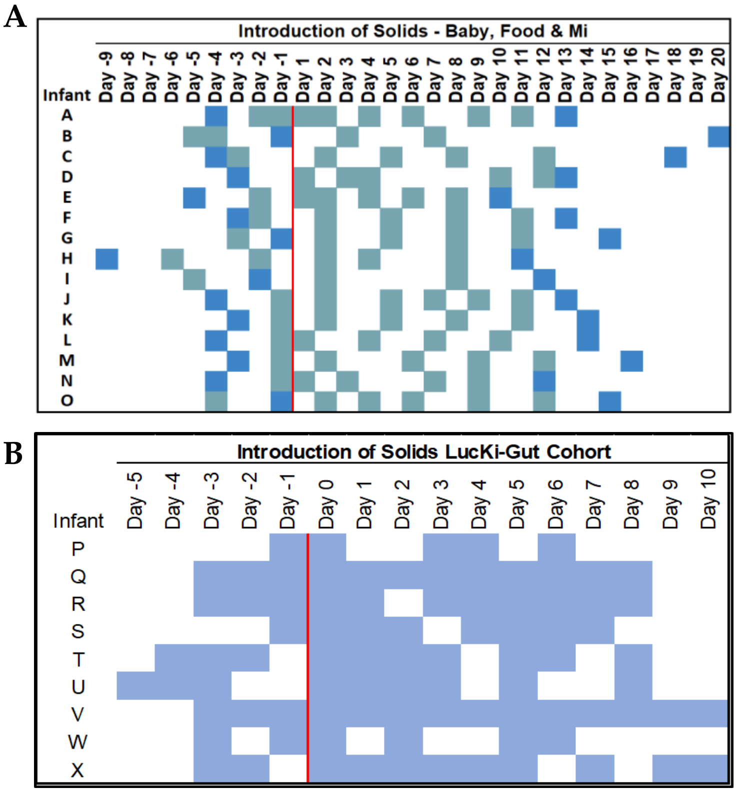

Figure S1: Log of sample collection over the study period at the time of solid food introduction. Colored squares indicate a sample was collected on that day. Day 1 is the first day where solid foods were introduced. **A)** Baby, Food & Mi; dark blue squares indicate fresh samples, blue-green squares indicate frozen samples. **B)** LucKi-Gut.

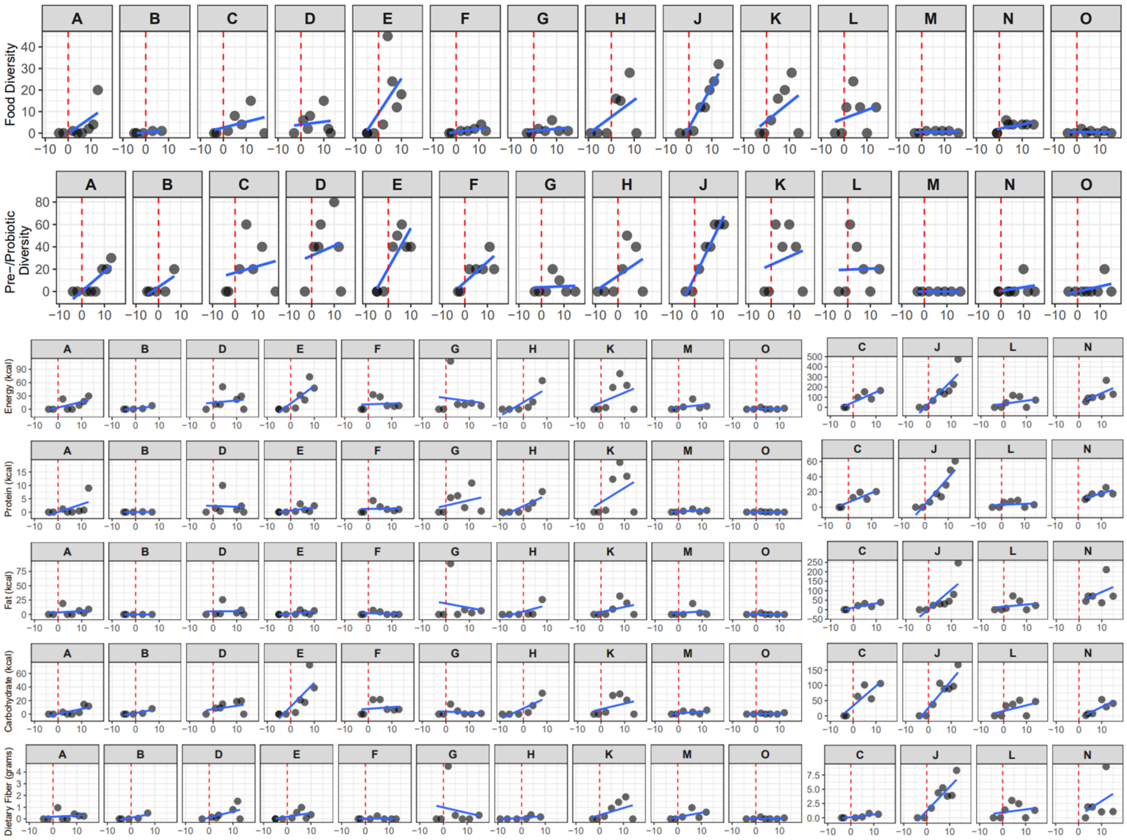

Figure S2: Individual infant (A - O) daily dietary intake in the Baby, Food & Mi sub-study for the macronutrients, fiber, and the dietary diversity scores. For the macronutrients and fiber, there was a delineation between infants with high or low caloric intake. High caloric infants: C, J, L, N.

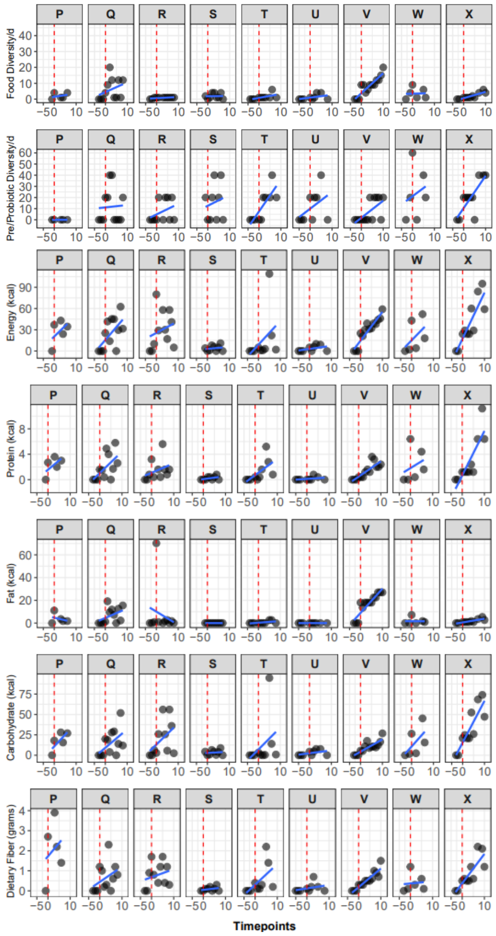

Figure S3: Dietary intake over the study period in the LucKi-Gut study on a daily basis for the macronutrients, fiber, and the dietary diversity scores by infant.

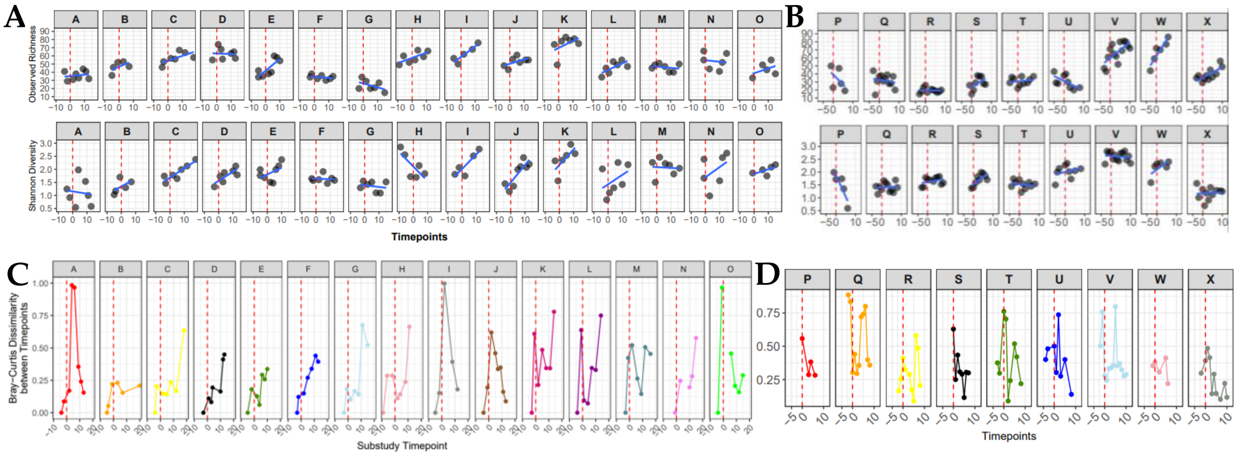

Figure S4: Alpha and Beta Diversity Metrics on a day-to-day basis for both cohorts. **A)** Observed richness and Shannon diversity over time for each infant of the Baby, Food & Mi study individually. **B)** Observed richness and Shannon diversity over time for each infant of the LucKi-Gut study individually. **C)** Changes in Bray-Curtis dissimilarity from sample to sample over the study period for infants of the Baby, Food & Mi study. **D)** Changes in Bray-Curtis dissimilarity from sample to sample over the study period for infants of the LucKi-Gut study.

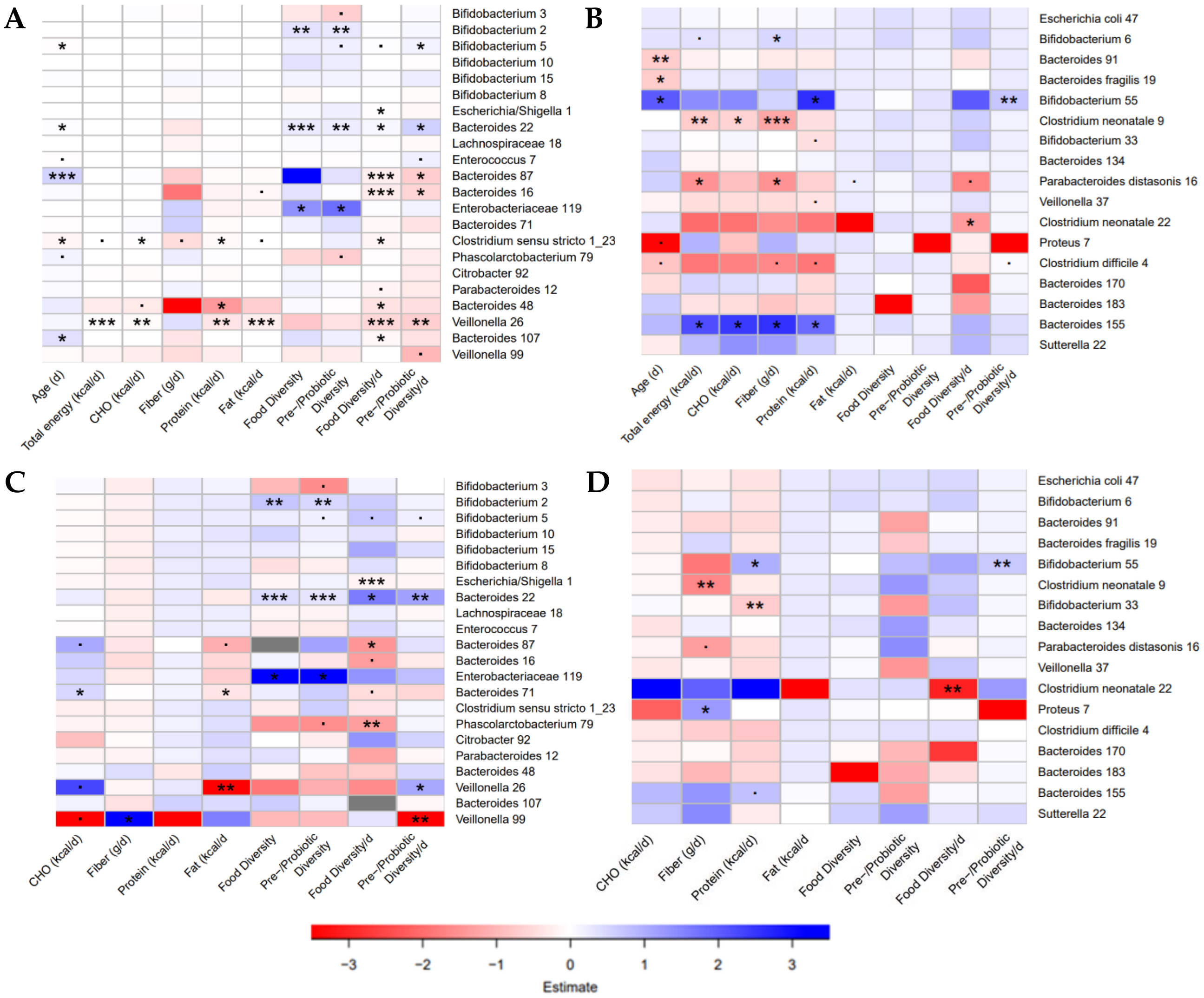

Figure S5: Heatmaps of the associations between bacterial ASVs and dietary variables, as analyzed with negative binomial regression models. **A)** Univariate models of the Baby, Food & Mi study. **B)** Univariate models of the LucKi-Gut study. **C)** Multivariate models of the Baby, Food & Mi study corrected for total caloric intake. **D)** Multivariate models of the LucKi-Gut study corrected for total caloric intake. .: $p < 0.1$, * : $p < 0.05$, ** : $p < 0.01$, *** : $p < 0.001$.

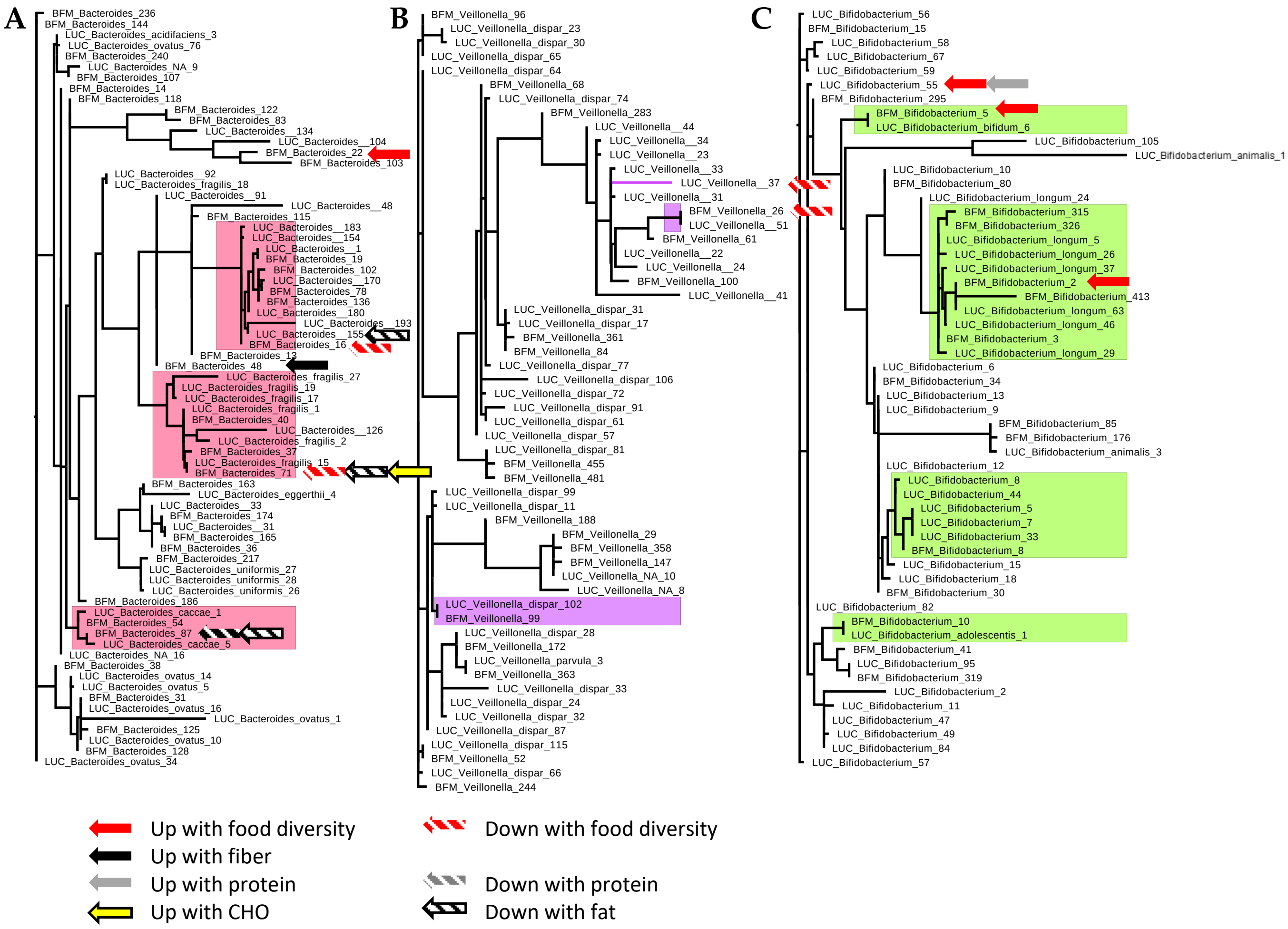

Figure S6: Alignment trees for the bacterial genera of interest from the results of the negative binomial regressions for the ASVs from both cohorts. Colored branches indicate a high probability of the ASVs belonging to the same bacterial species. **A**: *Bacteroides*, **B**: *Veillonella*, **C**: *Bifidobacterium*. Arrows indicate an association or trend with nutrition.
